# Correction: The effectiveness and efficiency of using normative messages to reduce waste: A real world experiment

**DOI:** 10.1371/journal.pone.0303709

**Published:** 2024-05-09

**Authors:** Gabby Salazar, João Neves, Vasco Alves, Bruno Silva, Jean-Christophe Giger, Diogo Veríssimo

In Table 3, the headings for the third and fourth columns should be reversed. The column heading on the third column should be "No drinks sold" and the column heading on the fourth column should be "No of straws taken”. Please see the correct Table 3 here.

**Table 3:** Mean ratio of straws taken to drinks sold for two message conditions and a control.

Condition	N^o^ days data collected	N^o^ drinks sold	N^o^ straws taken	Ratio of straws taken to drinks sold $\bar{x}$ (SD)
Positive injunctive social norm	25	3888	431	0.111 (0.045)
Negative injunctive social norm	26	3611	548	0.152 (0.076)
Control	26	3847	618	0.172 (0.094)
